# Evaluation of SHAPE cognitive therapy coaching for PTSD and depression symptoms in healthcare workers repeatedly exposed to trauma

**DOI:** 10.1038/s41598-026-36057-5

**Published:** 2026-01-17

**Authors:** Jennifer Wild, Aimee McKinnon, Abbie Wilkins, Ceri Storch, Haddi Browne, Anke Ehlers

**Affiliations:** 1 Department of Experimental Psychology, University of Oxford, Oxford, UK; 2 Phoenix Australia Centre for Posttraumatic Mental Health, Department of Psychiatry, University of Melbourne, Melbourne, Australia; 3 Oxford Health NHS Foundation Trust, Sandford Road, Littlemore, Oxford, UK; 4 Department of Psychology, University of Exeter, Exeter, UK; 5 Department of Psychology, University of Bath, Bath, UK

**Keywords:** Healthcare worker, COVID-19, Posttraumatic stress disorder, Depression, Cognitive therapy, SHAPE coaching intervention, Psychology, Health care

## Abstract

Healthcare workers are at increased risk of posttraumatic stress disorder (PTSD), depression and generalised anxiety disorder. Evidence-based interventions tailored to this workforce are limited. We developed and evaluated a telephone-based cognitive therapy coaching intervention targeting PTSD and depression. One hundred three healthcare workers seeking support during the COVID-19 pandemic completed measures at baseline, after three-week symptom monitoring, following six-week coaching, and at three-month follow-up. Sixty-five participants met criteria for probable PTSD (PCL-5 ≥ 32) or probable depression (PHQ-9 ≥ 10), and 38 experienced subthreshold symptoms. For PTSD (PCL-5) and depression (PHQ-9), reliable recovery rates increased substantially from symptom monitoring (PTSD: 14.6%; depression: 15.8%) to intervention (PTSD: 77.1%; depression: 64.3%), with sustained recovery at three months (68.8% and 52.6%, respectively). Overall, rates of reliable improvement increased from 41.1% to 65.8% for PTSD and from 20.0% to 63.1% for depression during intervention, with gains maintained at follow-up (72.2% and 56.0%, respectively). Mean PTSD, depression and anxiety (GAD-7) scores fell below clinical thresholds post-intervention and remained below at follow-up. Intervention-specific effects were observed for sleep, resilience, and wellbeing, with sustained improvements at follow-up. Cognitive therapy coaching is a promising intervention for healthcare workers with PTSD and depression symptoms. A randomised controlled trial is warranted.

**Supplementary Information:**

The online version contains supplementary material available at 10.1038/s41598-026-36057-5.

## Introduction

Healthcare workers devote their working lives to treating illnesses and facilitating patient care, often facing exposure to potentially traumatic events. Research demonstrates that occupational trauma, such as witnessing serious injury or death, is just as frequently associated with posttraumatic stress disorder (PTSD) in this workforce as non-occupational trauma (4). Notably, a significant proportion of healthcare workers report abuse and neglect in their childhoods^1–3^. The cumulative exposure to traumatic events, coupled with occupational pressures, including staffing challenges, irregular shifts and time-related stressors^4^, elevates risk for trauma-related psychopathology in this workforce. Studies using structured diagnostic interviews have reported prevalence rates of 14.3% for generalised anxiety disorder, 7.9% to 44% for PTSD, and 13.7% to 39% for major depression among healthcare workers^3, 5^. Discrepancies in rates are likely due to sampling methods and whether participants were predominantly patient-facing or non-clinical healthcare workers. Although rates of mental health problems are high, treatment seeking is low, with healthcare workers reporting stigma and lack of time as barriers to care^6^. Digitally-enabled interventions may improve accessibility for this workforce.

Several digital interventions have been developed or adapted to address psychological distress in healthcare workers. A multicentre randomised controlled trial (*n* = 155) of MyHealthToo, an 8-week online CBT program, found no between-group differences on the primary outcome of perceived stress compared to an active control (bibliotherapy)^7^. However, the intervention demonstrated significant reductions in work-related rumination at post-treatment and posttraumatic stress symptoms at one-month follow-up, with reductions in rumination mediating PTSD symptom improvement. RESTORE (Recovering from Extreme Stressors Through Online Resources and E-health), an 8-module self-directed CBT-based intervention for anxiety, depression, and PTSD associated with COVID-19-related traumatic and extreme stressors, was evaluated in an uncontrolled trial with 21 healthcare workers, demonstrating large effect sizes (0.84–1.58)^8^. Among controlled trials with follow-up assessments, three interventions demonstrated evidence of effectiveness: two mobile wellbeing apps used for four weeks^9^; a 12-week mobile wellness program combining physical activity tracking, online exercise sessions, and health coaching^10^; and a two-week daily Tai Chi program^11^. Notably, a well-powered randomised controlled trial (*n* = 482) of PsyCovidApp, a CBT and mindfulness based app, targeting skills to manage emotions, healthy lifestyle behaviours, burnout, and social support, found no significant differences between intervention and control groups on primary outcomes^12^. While subgroup analyses showed that healthcare workers receiving concurrent psychotherapy or psychotropic medication who used the app experienced improvements in composite scores of depression, anxiety, and stress symptoms, these findings suggest that improvements may have been primarily attributable to the concurrent treatments rather than the app itself.

Broader evidence reviews of interventions for healthcare workers highlight methodological limitations in existing research and important patterns regarding intervention effectiveness. A comprehensive evidence synthesis of interventions to support healthcare workers’ mental health during pandemics identified significant methodological limitations across 27 papers examining interventions adapted or developed during COVID-19, including variable study quality, small sample sizes, lack of control conditions, heterogeneous designs, and largely descriptive research^13^. A systematic review of psychosocial interventions for disaster-exposed healthcare workers found that while evidence-based CBT approaches for PTSD and anxiety demonstrated reliable symptom improvements, single-session debriefing and psychological first aid workshops showed limited efficacy^14^. These findings highlight the need for interventions that balance efficacy with accessibility, and suggest that a minimum dose is likely to be greater than one session.

Whilst first-line digital treatments for PTSD and comorbidity are becoming more widely available and reduce therapist time, patients typically need to commit similar amounts of time as they would for face-to-face trauma-focused therapy^15, 16^. There is therefore a need for tailored evidence-based interventions that can be delivered flexibly and for which session length and out-of-session requirements may be briefer than existing in-person or digitally-enabled approaches.

Coaching is a result-oriented, systematic process in which the coach facilitates the attainment of the client’s goals^17^. More recently, coaching programmes (e.g., BetterUp) have been developed to improve mental health, wellbeing and response to stress. Coaching calls can be briefer than traditional therapy sessions, with research showing that the most common session length ranges from 30 to 39 min^18^ compared to the standard 55 to 90 min for trauma-focused CBT for PTSD therapy sessions. Additionally, coaching appears to be associated with less stigma than psychotherapy^19^. A meta-analysis of workplace coaching interventions demonstrate medium effect sizes for improvements in wellbeing, coping, performance, work attitudes and goal attainment^20^. It is unclear whether a coaching format that primarily draws on cognitive therapy for PTSD would be acceptable for healthcare workers and to what extent the coaching intervention may be associated with PTSD and depression symptom reduction.

This study describes the development and evaluation of a cognitive therapy coaching intervention for PTSD and depression developed during the COVID-19 pandemic for healthcare workers. The intervention, called SHAPE (Supporting Healthcarel and Paramedic Employees with cognitive therapy coaching), was derived from first-line trauma-focused cognitive therapy for PTSD (CT-PTSD)^21^ and rumination-focused CBT for depression (RF-CBT)^22^. In this pilot study, SHAPE is evaluated with *N* = 103 healthcare workers who requested intervention during the COVID-19 pandemic for symptoms of PTSD or depression or both and consented to receive a telephone-based coaching approach. Healthcare workers were required to wait three weeks before accessing the intervention. During this wait period, they were asked to complete weekly questionnaires assessing PTSD and depression symptoms. This pilot study compares the change achieved during the intervention to the change achieved during the monitoring control period. We hypothesised that symptom improvement in PTSD and depression during the six-week coaching intervention would exceed changes observed during the monitoring phase, and that gains would be sustained at three-month follow-up. We selected PTSD and depression as primary outcomes because they are common sequelae of trauma exposure, frequently co-occur, share overlapping mechanisms and among particular subgroups of healthcare workers, such as paramedics, are substantially elevated compared to the general population^23, 24^. Secondary outcomes examined the broader impact of the intervention on generalised anxiety, insomnia, resilience and wellbeing. We hypothesised that the coaching intervention would be associated with improvements across these secondary outcomes.

Drawing on the Ehlers and Clark cognitive model of PTSD^25^ and cognitive theories of depression^26, 27^, we assessed cognitive and behavioural processes that may correlate with symptom change, including negative posttraumatic cognitions, maladaptive responses to intrusive memories and self-devaluative beliefs. We predicted that changes in posttraumatic cognitions would correlate with PTSD symptom reduction, controlling for baseline PTSD symptom severity, since these appraisals are central maintaining factors in Ehlers and Clark’s cognitive model of PTSD^25^, and latent growth curve analyses demonstrate that changes in these cognitive processes precede symptom reduction with CT-PTSD^28–30^. We further hypothesised that changes in maladaptive responses to intrusive memories would correlate with PTSD symptom reduction. For depression, we predicted that changes in rumination would correlate with symptom improvement, given that intrusive memories of negative life events are common in depression^31^, can influence the content of rumination^32^, and prospective studies demonstrate that rumination mediates the relationship between self-devaluative beliefs and subsequent depressive episodes^27, 33^. Mediation analyses further demonstrate that changes in rumination precede changes in depression symptoms^34^, that reducing rumination can prevent the onset of depression^35^ and mediate improvements in PTSD symptoms in healthcare workers^7^. Since SHAPE explicitly targets self-devaluative beliefs through CT-PTSD-informed techniques and rumination through rumination-focused CBT techniques, we hypothesised that changes in these processes would correlate with improvement in depression. Specifically, we predicted that changes in rumination as a response to intrusive memories would correlate with depression symptom reduction, and that changes in self-devaluative beliefs would correlate with improvement in depression when controlling for baseline depression severity.

## Methods

### Participants

Participants were healthcare workers who had delivered or directed care for patients with COVID-19, recruited from ten National Health Service (NHS) and Ambulance Service Trusts in England and Scotland, six of whom were partnered with NHS staff Wellbeing Hubs. Healthcare workers were informed of the study via email, flyer, radio interviews, and through Wellbeing Hub communications. Interested individuals self-referred to request the intervention. In partnered hubs, the study team provided training and supervision for staff to offer cognitive therapy coaching. Participants were eligible if they were experiencing primary PTSD or depression symptoms, regardless of whether they met diagnostic thresholds on standardised measures. Exclusion criteria included active risk behaviour or intent and a primary presenting problem other than PTSD or depression. See Table 1 for sample demographics and clinical characteristics. Ethics and protocol approval were granted by the University of Oxford Medical Sciences Interdivisional Research Ethics Committee (MSD-Ethics). All methods were carried out in accordance with MSD-Ethics guidelines, regulations and approved procedures. All participants provided informed consent.

Participants were eligible if their self-reported primary concerns during the clinical interview centred on traumatic stress reactions (e.g., re-experiencing symptoms, hypervigilance, trauma-related distress) and/or depressive symptoms (e.g., low mood, hopelessness), regardless of whether they met diagnostic thresholds on standardised measures. For participants presenting primarily with PTSD symptoms, intrusive memories were required to relate to one or up to two discrete traumatic events or several traumatic episodes during a longer period of high threat, such as domestic abuse, childhood abuse, COVID-19 or prolonged work-related trauma. This inclusive approach was adopted because the intervention was delivered as frontline mental health support during the COVID-19 pandemic to provide accessible care for distressed healthcare workers and, where applicable, to prevent subthreshold symptoms from progressing to diagnostic status. As a result, some participants reported no direct traumatic events but nonetheless presented with clinically meaningful depressive symptoms, while others reported multiple traumatic exposures and severe PTSD symptoms. This approach ensured that the sample reflected the diverse psychological presentations of healthcare workers during the pandemic.

Exclusion criteria were: (1) active suicidality or risk to others requiring immediate crisis intervention, (2) primary mental health presentation other than PTSD or depression (e.g., eating disorder), and (3) explicit preference for standard psychological therapy rather than the coaching format.

### Measures

Measures are categorised into assessment of trauma exposure, primary and secondary outcomes and proposed cognitive processes associated with symptom change.

#### Assessment of trauma exposure

The Life Events Checklist (LEC) was used to assess participants’ exposure to a range of potentially traumatic events, including childhood adversity related to childhood emotional abuse, neglect and physical abuse^36^.

#### Primary outcomes

PTSD symptoms were assessed using the PTSD Checklist for DSM-5 (PCL-5)^37^, a 20-item self-report measure assessing symptoms across four domains: re-experiencing, avoidance, negative alterations in cognition and mood, and hyperarousal. Items are scored on a 5-point scale (0 = Not at all to 4 = Extremely), with total scores ranging from 0 to 80. Scores of 32 or higher indicate probable PTSD. Internal consistency in the sample was excellent (Cronbach’s alpha = 0.94). Depressive symptoms were assessed using the Patient Health Questionnaire-9 (PHQ-9)^38^, a 9-item self-report measure of depressive symptom severity. Items are scored on a 4-point scale (0 = Not at all to 3 = Nearly every day) with total scores ranging from 0 to 27. Scores of 10 or higher indicate probable depression. Internal consistency in the sample was good (Cronbach’s alpha = 0.89).

#### Secondary outcomes

The Generalized Anxiety Disorder Questionnaire (GAD-7)^39^ is a 7-item measure that assesses symptoms of anxiety with items scored from 0 (Not at all) to 3 (Nearly every day). Scores range from 0 to 21. A score of 8 or higher suggests probable anxiety disorder. Internal consistency in the sample was good (Cronbach’s alpha = 0.88). The Insomnia Severity Index^40^ is a 7 question measure of sleep quality. Scores range from 0 to 28, with higher scores indicating poorer quality of sleep. Internal consistency in the sample was excellent (Cronbach’s alpha = 0.91). The Warwick-Edinburgh Mental Wellbeing Scale (WEMWBS)^41^ is a 14-item measure of wellbeing. Items are scored on a 5-point scale (1 = None of the time to 4 = All of the time) with total scores ranging from 14 to 70. Higher scores indicate greater wellbeing. Internal consistency in the sample was excellent (Cronbach’s alpha = 0.95). The 10-item Connor Davidson Resilience Scale^42^ is a measure of resilience, with items scored on a 5-point scale (0 = Not true at all to 4 = True nearly all the time). Total scores range from 0 to 40 with higher scores indicating greater resilience. Internal consistency in the sample was excellent (Cronbach’s alpha = 0.91).

#### Proposed cognitive processes associated with symptom change

The Responses to Intrusions Questionnaire (RIQ)^43^ is a 19-item measure that assesses unhelpful responses to intrusive memories which include suppression (items 1–6; Cronhbach’s alpha = 0.89) rumination (items 7–14; Cronbach’s alpha = 0.85), and numbing (items 15–19; Cronbach’s alpha = 0.74). Items are measured on a 4-scale (0 = Never to 3 = Always). Total scores for the scale range from 0 to 57, with higher scores indicating more frequent use of maladaptive responses to unwanted memories. The Posttraumatic Cognitions Inventory (PTCI)^44^ is a 20-item short form^28^ of the PTCI^44^, a self-report measure that assesses excessively negative appraisals of the trauma, with items rated on a 7-point scale (1 = Totally disagree to 7 = Totally agree). Total scores range from 20 to 140 (Cronbach’s alpha = 0.94) with higher scores representing greater endorsement of posttraumatic negative appraisals. Self-devaluative appraisals are measured by the sum of items 12, 15 and 16 (‘I am inadequate,’ ‘There is something wrong with me as a person’ and ‘I feel dead inside’). Internal consistency for this subscale was good (Cronbach’s alpha = 0.82).

### Design

This study employed a repeated measures within subjects design. The full set of symptom and process measures were administered at four time points: baseline (T1), three weeks later at post-monitoring/pre-intervention (T2), after six weeks of coaching at post-intervention week 9 (T3), and at three month follow-up, week 21 (T4).

### Procedure

**Fig. 1:**
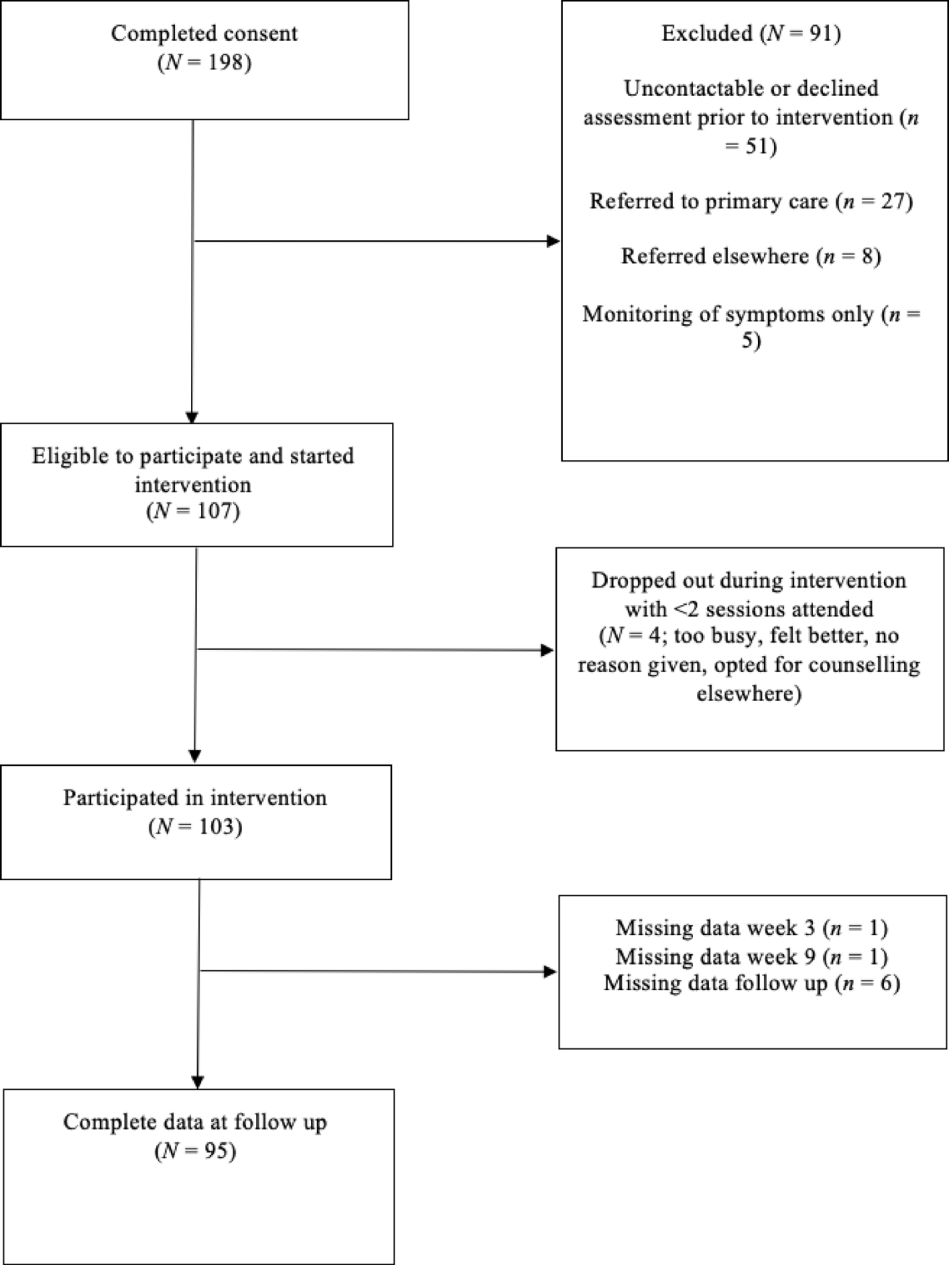
Recruitment and retention flow diagram.

Recruitment took place in England and Scotland from August 2020 to August 2022. Participants were informed of the study via email, flyer, radio interview, and advertisement at staff team meetings. All contact for the study was remote and delivered via telephone, email, and online questionnaires. Participants completed consent and all questionnaires online via Qualtrics. Suitability assessments were carried out by a clinical psychologist or psychological wellbeing practitioner following completion of the baseline questionnaires. Participants were offered the intervention, a referral to their local talking therapies service, or other appropriate service depending on presenting problem and participant preference. Figure 1 illustrates the recruitment and retention flow diagram.

### SHAPE development and overview

SHAPE was developed during the COVID-19 pandemic, primarily drawing on cognitive therapy for PTSD^21^, a first-line intervention for PTSD recommended by the National Institute for Health and Care Excellence. Additionally, SHAPE drew on the PREVENT-PTSD randomised controlled trial^45^, research on modifiable risk factors for depression and PTSD in healthcare workers^46^, and IF-THEN plans for rumination, drawn from rumination-focused cognitive therapy for depression^47^. The SHAPE intervention had three aims. First, it aimed to provide timely and accessible support for healthcare workers affected by COVID working. Second, it was developed to target cognitive and behavioural maintaining factors, including rumination, unwanted memories, behavioural disengagement, and negative appraisals. Third, it aimed to address trauma symptoms without the reliving or prolonged exposure aspects of trauma-focused therapy. SHAPE coaching differs from CBT delivered as psychotherapy in two ways. First, SHAPE coaching prioritises skill development, such as trigger discrimination for re-experiencing symptoms, rather than employing more intensive therapeutic techniques, such as reliving. Second, coaches are not required to be formally trained CBT practitioners; they could be psychology graduates who receive structured training and supervision in SHAPE coaching.

### Treatment targets

SHAPE draws on the three treatment targets of CT-PTSD^21^. The first target focuses on reducing PTSD re-experiencing symptoms. This is achieved through stimulus discrimination as the primary memory-focused technique, which helps reduce re-experiencing and overgeneralised responses to triggers in the present that share overlapping sensory features with past traumatic experiences. Stimulus discrimination involves directing attention to all of the differences between a trigger in its present context and the past trauma^21^. Supplement 1 outlines core differences in trauma-focused trigger discrimination between SHAPE and CT-PTSD, with Supplementary Table S1 providing an example of Then vs. Now discrimination in SHAPE.

The second target involves modifying negative appraisals, which is relevant for PTSD and depression. This is accomplished through cognitive restructuring that draws on the outcomes of behavioural experiments, responsibility pie charts, and surveys. The third target addresses maintaining strategies for PTSD and depression. Behavioural experiments are used to address strategies, such as scanning for danger or overprotecting close ones, that maintain a sense of threat. IF-THEN plans^22^ are employed to address rumination by identifying signs of rumination that span a number of domains (such as situations, physical signs, behaviours, and feelings) and developing action plans to execute in response to the early signs of rumination. The intervention incorporates a reclaiming life approach with feasible daily breaks to address low mood, mindful of healthcare workers’ full schedules and reported fatigue. Supplementary Table S2 provides an overview of the treatment tools used in SHAPE.

SHAPE consists of six telephone sessions, each lasting 20–40 min, delivered over a period of 6–8 weeks. Participants also have access to six interactive online modules, each requiring 20–40 min to complete. Supplementary Table S3 describes the online learning modules. The focus of each session is determined by weekly symptom measures. Every call includes experiential exercises that are completed with a coach and then continued independently by the participant between sessions. To provide timely intervention, the symptom monitoring period was limited to three weeks prior to the start of the intervention.

### Intervention delivery

The intervention was initially delivered by practitioners in the study team (a clinical psychologist, psychological wellbeing practitioner, and a research assistant; 81% of the sample received the intervention from the study team) and later by trained staff from NHS Wellbeing Hubs (clinical and counselling psychologists, assistant psychologists, psychological wellbeing practitioners, and mental health nurses). Each participant was assigned to a single coach for the duration of the intervention. Participants were allocated to a coach based on coach availability at the time of assessment. The study team supervised the delivery of the intervention by the hubs and supervision of the study team was conducted by the study lead on a weekly basis.

To promote intervention fidelity, several procedures were implemented across four domains: training, the structured SHAPE protocol, supervision and session documentation. All coaches completed one-day training in the SHAPE (Supporting Healthcare and Paramedic Employees) coaching protocol prior to study commencement, were required to treat two training cases prior to delivering SHAPE as part of the study, and to attend weekly supervision throughout intervention delivery. Coaches followed the structured SHAPE protocol, which specified session content based on weekly symptom scores and outlined core tools to be delivered, which were based on CT-PTSD and IF-THEN plans for rumination drawn from RF-CBT. All coaches attended weekly supervision with a clinical psychologist or psychological wellbeing practitioner trained in CT-PTSD and IF-THEN plans for rumination. Supervisors received weekly supervision from the study lead, a clinician with extensive training in both approaches and experience delivering these interventions across multiple RCTs. Supervision included case discussion, review of participant outcome measures, problem-solving challenges in the delivery of the intervention, role plays, and guidance on appropriate implementation of intervention tools. Following each session, supervisors emailed coaches an individualised agenda for each participant’s next call, specifying which intervention component to implement based on current symptom scores. Coaches completed a brief session checklist after each session documenting protocol components and skills covered. These checklists were reviewed during supervision to monitor adherence and provide feedback on intervention delivery.

### Data analysis

We conducted an a priori sensitivity analysis indicating that a sample of *N* = 88 would provide adequate power (90%) to detect a small effect size of f = 0.14 with four repeated measurements (α = 0.05). Accounting for up to 15% attrition, we recruited *N* = 103 healthcare workers.

As the sample included both those above and below clinical cut offs, i.e. those who met clinical caseness for depression or PTSD and those who did not, testing was conducted on the full sample, whereas recovery rates are only reported for the subgroup who were above clinical cut off. Repeated measures analysis of variance was conducted to investigate PTSD and depression symptom change over time. There were four time points: baseline (T1), pre-intervention (T2) which occurred at week 3 after assessment and monitoring, post-intervention (T3) which occurred at week 9 after six weeks of coaching, and three month follow-up (T4) which occurred 21 weeks after T1. Bonferroni corrected (*p* < .02) planned pairwise comparisons were conducted to compare the mean change in PTSD and depression scores at the different time points. Missing data values for the PCL-5 and PHQ were low (7.69% of participants representing 1.92% of total data across the four time points) and missing at random (Little’s MCAR test *p* = .494) thus no multiple imputation was used. One participant did not complete measures at week three, one did not complete them at week nine, and six were missing at follow-up.

To analyse hypotheses two and three investigating proposed correlates of symptom reduction, we conducted partial correlations between pre- to post-intervention change scores for hypothesised processes (suppression, rumination, numbing, posttraumatic cognitions) and symptom outcomes (PTSD and depression), controlling for pre-intervention symptom severity. While this approach cannot establish that changes in cognitive processes preceded and caused symptom improvements, it provides preliminary evidence of covariation between cognitive processes and symptom change during treatment. This approach is consistent with exploratory methods used in pilot studies that examine whether changes in psychological processes are associated with improvements in PTSD and depression symptoms during treatment^48^.

Reliable improvement and reliable recovery were reported across three study phases: Baseline to Week 3 (Monitoring), Week 3 to Week 9 (Intervention), and Baseline to Follow-up (Sustained Gains). Reliable improvement was defined as a difference of ≥ 6 for the PHQ-9 and ≥ 10 on the PCL-5 (49) and was only reported for individuals for whom baseline scores were above these measurement error values. Reliable recovery was defined following the UK NHS Talking Therapies for anxiety and depression manual (version 7). An individual was classified as having reliably recovered from PTSD if they scored below clinical cut-offs on both the PCL-5 and PHQ-9 at the end of the time interval, showed improvement exceeding the reliable change index on the PCL-5, and did not reliably deteriorate on either measure. Reliable recovery from depression required scores below clinical cut-offs on both the PHQ-9 and GAD-7 at the end of the time interval, improvement exceeding the reliable change index on the PHQ-9, and no reliable deterioration on either measure^49^. Denominators for recovery rates represent participants above clinical threshold on the respective measure at the start of each period (PCL-5 ≥ 32 for PTSD; PHQ-9 ≥ 10 for depression). Rates of reliable deterioration are also reported. Data were assessed for normality prior to analysis. Non-normal variables were square root transformed to meet parametric test assumptions, resulting in transformation of the WEMWBS.

## Results

### Sample characteristics

Sample demographics and characteristics are displayed in Table 1. The number of calls attended, modules completed, and completion times are presented in Table 2. Pre- and post-intervention scores for symptom and process measures are displayed in Table 3. At baseline, 55.3% (*N* = 57) of the sample were above clinical caseness on the PHQ-9; 46.6% (*N* = 48) were above clinical threshold on the PCL-5; 38.8% (*N* = 40) were above clinical cut off on both measures, and 63.1% (*N* = 65) were above cut off on one or both measures. The time since the index event ranged from one month to 56 years (Median = 18 months, IQR = 6–96). There was no association between reliable recovery or improvement and gender or trauma type (occupational versus personal; *p*s > 0.241).

**Table 1:** Sample and index trauma event characteristics.

Variable	Total sample (*N* = 103)
Gender	71.8% female (74)
Age	38.73 (9.98)
Ethnicity	
White British	82.5% (85)
Other white	7.8% (8)
Black British	2.9% (3)
Asian	1.8% (2)
Other mixed	1.8% (2)
Other/not specified	2.9% (3)
Job role	
Nurse	38.8% (40)
Ambulance staff	30.1% (31)
Allied health	21.4% (22)
Clinical support	3.9% (4)
Medical doctor	2.9% (3)
Non-clinical	1.9% (2)
Clinical hub	1.9% (1)
No history of mental health treatment	51.5% (53)
Index trauma event	
Event type	
Severe human suffering	21.4% (22)
Life threatening illness/injury	20.4% (21)
Sudden violent death	11.7% (12)
Childhood abuse	8.7% (9)
Sudden accidental death	7.8% (8)
Sexual assault	7.8% (8)
Traffic accident	4.9% (5)
Assault	3.9% (4)
Caused serious harm	1.9% (2)
Natural disaster	1% (1)
Other event	10.7% (11)
Occupational or personal trauma	53.4% Personal (55)
Trauma unrelated to COVID-19	72.8% Unrelated (75)
PTSD symptoms (PCL-5): mean (SD) [range]	30.78 (19.01) [0–67]
Depression symptoms (PHQ-9): mean (SD) [range]	10.63 (6.53) [0–25]

### Assessment of trauma exposure

Total traumas reported ranged from 0 to 16, (*M* = 8.05, *SD* = 4.47), with traumas experienced as part of a person’s job ranging from 0 to 15 (*M* = 4.77, *SD* = 4.85). 35% of the sample reported an experience of childhood trauma (emotional abuse, physical abuse, sexual abuse, and/or neglect).

### Intervention engagement

Participants attended a mean number of 5.83 (SD = 0.76) calls. Module completion rates and median completion times are presented in Table 2. Completion times were positively skewed and included periods of participant inactivity, when the module was open but idle. Because of this, median values and interquartile ranges (IQRs) are reported. Median completion times ranged from approximately 22 to 36 min, suggesting comparable engagement durations across modules, although some participants remained in modules substantially longer. Variation in completion times is also likely to reflect differences in module content.

**Table 2:** Module completion rates %(n) and median completion Times.

	Habits and dwelling: how to change them	Dealing with unwanted memories: then vs. now	Get out of your head with helpful thinking	Attention training: it matters what you focus on	Transforming worries and planning ahead	Dealing with guilt and self-blame
Percent completion (*N* = 103)	91.3% (94)	80.6% (83)	73.8% (76)	73.8% \(76)	57.3% (59)	42.7% (44)
Median completion time, mins	23.1 (6.9–51.9)	30.2 (16.7–131.1)	36.5 (7.8–67.3)	21.8 (2.5–94.9)	28.0 (2.4–144.5)	26.5 (12.3–67.7)

IQR = Interquartile range.

### Primary outcomes

A repeated measures ANOVA with Greenhouse-Geisser correction demonstrated that mean PTSD symptom severity scores differed significantly between time points (F(1.79, 168.30) = 90.59, *p* < .001, partial η² = 0.49), indicating a large effect size. Contrasts revealed significant reductions in PCL-5 scores from baseline to end of monitoring/pre-intervention (F(1, 94) = 22.30, *p* < .001, partial η² = 0.19) and from end of monitoring/pre-intervention to end-of-intervention (F(1, 94) = 93.60, *p* < .001, partial η² = 0.50). No significant differences were found between end of intervention and 3 months follow-up (F(1, 94) = 1.87, *p* = .174, partial η² = 0.02), indicating that gains were maintained during the follow-up period.

For depression symptom severity scores, repeated measures ANOVA with Greenhouse-Geisser correction revealed a statistically significant difference between time points (F(1.84, 172.62) = 67.18, *p* < .001, partial η² = 0.42), demonstrating a large effect size. Contrast analyses showed significant decreases in PHQ-9 scores from baseline to end-of-monitoring/pre-intervention (M = 8.62, SD = 6.43; F(1, 94) = 21.67, *p* < .001, partial η² = 0.19) and from end-of-monitoring/pre-intervention to end-of-intervention, F(1, 94) = 62.77, *p* < .001, partial η² = 0.40). However, a small but significant increase in PHQ scores was observed from end-of-intervention to three months follow-up F(1, 94) = 6.90, *p* = .010, partial η² = 0.07).

**Table 3:** Means and standard deviations for symptom severity scores and process measures at each time point for the full sample (*N* = 103).

Measure	Time 1 Baseline Mean (SD)	Time 2 (3 weeks) Post-monitoring/pre-intervention Mean (SD)	Time 3 (9 weeks) Post-intervention Mean (SD)	Time 4 (21 weeks) 3-month follow-up Mean (SD)
Primary outcomes				
PCL-5	29.93 (18.99)	24.58 (17.79)	10.84 (9.12)	11.79 (9.99)
PHQ-9	10.39 (6.5)	8.62 (6.42)	4.3 (3.8)	5.02 (4.49)
Secondary outcomes				
GAD-7	9.98 (5.51)	8.87 (5.40)	4.19 (3.37)	4.77 (4.27)
ISI	11.72 (6.95)	11.27 (7.13)	7 (4.87)	7.40 (5.58)
WEMWBS	34.77 (10.60)	33.66 (5.04)	36.29 (5.04)	36.18 (5.30)
CD-RISC	22.84 (8.41)	23.81 (7.92)	27.54 (6.43)	27.12 (7.54)
Proposed cognitive processes associated with symptom change				
PTCI	58.91 (24.21)	57.27 (24.92)	45.4 (20.18)	44.08 (20.89)
Suppression	9.38 (4.66)	9.07 (4.39)	6.77 (3.85)	6.37 (4.24)
Rumination	8.89 (5.7)	8.44 (6.24)	5.18 (3.92)	5.31(4.09)
Numbing	3.88 (3.01)	4.09 (3.27)	2.46 (2.20)	2.85 (2.59)
Self-devaluative beliefs	9.49 (5.03)	9.18 (5.01)	6.84 (4.00)	6.65 (4.12)

PCL-5 = Posttraumatic Stress Disorder Checklist for DSM-5; PHQ-9 = The Patient Health Questionnaire Depression Scale; GAD-7 = Generalized Anxiety Disorder Scale; ISI = Insomnia Severity Index; WEMWBS = Warwick-Edinburgh Mental Wellbeing Scale; CD-RISC = Connor-Davidson Resilience Scale; RIQ = Responses to Intrusions Questionnaire; PTCI = Posttraumatic cognitions inventory.

The results demonstrate that PTSD and depression symptoms change over time from baseline to post-intervention and that for PTSD, gains are maintained at follow-up. For depression, a small but significant increase in scores was observed between end-of-coaching and three-month follow-up although scores continued to be well below clinical threshold at follow-up. Consistent with hypothesis one, the symptom change with the intervention was greater than the change observed during the monitoring period.

### Reliable Improvement, Recovery, and deterioration

Reliable improvement, remission, and reliable recovery are reported in Table 4. For PTSD symptoms, 68.8% of the sample who were above clinical cut off at baseline had met reliable recovery criteria by the end of the intervention and maintained recovery at follow up. Sustained reliable recovery was 52.6% for depression. Reliable deterioration in trauma or depression symptoms occurred for eight individuals during the assessment and monitoring period, one individual reliably deteriorated in trauma and depression during the intervention and one participant saw reliable deterioration in depression during follow-up.

**Table 4:** Reliable improvement, remission, and reliable recovery.

	Baseline to week 3 (monitoring)	Week 3 to week 9 (intervention)	Baseline to follow-up (sustained gains)
Full sample Reliable improvement^a^			
PTSD (PCL-5; baseline *n* = 90)	41.1% (37/90)	65.8% (52/79)	72.2% (65/90)
Depression (PHQ-9; baseline *n* = 75)	20.0% (15/75)	63.1% (41/65)	56.0% (42/75)
Above cut off sample Remission rates^b^			
PTSD (PCL-5; baseline *n* = 48)	27.1% (13/48)	88.6% (31/35)	81.3% (39/44)
Depression (PHQ-9; baseline *n* = 57)	33.3% (19/57)	83.3% (35/42)	73.7% (42/57)
Reliable recovery^c^			
PTSD (PCL-5; baseline *n* = 48)	14.6% (7/48)	77.1% (27/35)	68.8% (33/48)
Depression (PHQ-9; baseline *n* = 57)	15.8% (9/57)	64.3% (27/42)	52.6% (30/57)

Missing data were treated as not recovered/not changed. Missing at week 3 *n* = 1, missing at week 9 *n* = 1, missing at month 3 *n* = 4.

^a^Reliable improvement reported in those who scored above the reliable change index at the initial time point. PCL-5 reliable change index = 10 points; PHQ-9 reliable change index = 6 points.

^b^Remission rates report those who were above caseness at the start of the interval, and below at the end. Clinical cut-offs were PCL-5 < 32 and PHQ-9 < 10.

^c^Reliable recovery was calculated according to the United Kingdom National Health Service Talking Therapies for anxiety and depression Manual (NHS England, 2024)^49^. An individual was classified as having reliably recovered in PTSD if they scored below the clinical cut-offs on both the PCL-5 and PHQ-9 at the end of the time interval, showed an improvement greater than the reliable change index on the PCL-5, and did not reliably deteriorate on either measure. Reliable recovery in depression was defined using the same criteria, except that the GAD-7 was required to be below its clinical cut-off at the end time point.

PCL-5 = Posttraumatic Stress Disorder Checklist for DSM-5 (Weathers et al., 2013); PHQ-9 = Patient Health Questionnaire-9 (Kroenke et al., 2001); GAD-7 = Generalized Anxiety Disorder-7 (Spitzer et al., 2006).

### Secondary outcomes

#### Anxiety, sleep problems, resilience and wellbeing

Repeated measures ANOVAs with Greenhouse-Geisser correction revealed significant time effects for anxiety (F(2.15, 167.27) = 53.30, *p* < .001, partial η² = 0.41), insomnia severity (F(2.18, 170.18) = 26.22, *p* < .001, partial η² = 0.25), and resilience (F(2.17, 169.16) = 20.01, *p* < .001, partial η² = 0.20), all demonstrating medium-to-large effect sizes. Pairwise comparisons revealed a range of patterns across measures. Anxiety (GAD-7) decreased significantly during monitoring and intervention periods, with intervention-period improvement nearly three times greater. Insomnia severity (ISI) and resilience (CD-RISC) showed no change during monitoring but significant improvements during intervention, demonstrating intervention-specific effects. All gains were maintained at 3-month follow-up (ps > 0.10).

Wellbeing showed a different pattern. While the overall time effect on square-root transformed scores was not significant (F(1.89, 151.30) = 1.63, *p* = .201), planned comparisons revealed the hypothesised pattern with scores remaining stable during monitoring (*p* = .464), improving significantly during intervention (*p* = .014), and maintained at follow-up (*p* = .799).

### Proposed cognitive processes associated with symptom change

#### PTSD symptom severity

##### Responses to unwanted memories

Controlling for pre-intervention PTSD severity, reductions in the frequency of suppression in response to intrusive memories during the coaching intervention were significantly correlated with change in PTSD symptoms (*r* = .43, df = 84, *p* < .001). Reductions in rumination in response to unwanted memories were not significantly associated with changes in PTSD symptoms at post-intervention (*r* = .18, df = 84, *p* = .11). Changes in numbing as a response to unwanted memories during the intervention showed a trend toward being related to changes in PTSD symptoms (*r* = .18, df = 84, *p* = .09).

##### Posttraumatic cognitions

After controlling for pre-intervention PTSD severity, reductions in posttraumatic negative cognitions (measured with the PTCI total score) during the coaching intervention were significantly correlated with PTSD symptom change at post-intervention (*r* = .37, df = 84, *p* < .001).

#### Major depression symptom severity

Controlling for pre-intervention depression symptom severity, reductions in rumination (in response to unwanted memories) were not significantly associated with reductions in depression symptom severity (*r* = .09, df = 84, *p* = .42). Changes in self-devaluative appraisals were significantly correlated with reductions in depression symptom severity at post-intervention (*r* = .38, df = 84, *p* < .001).

## Discussion

This pilot study evaluated the effectiveness of a digitally-supported telephone coaching intervention, derived primarily from cognitive therapy for PTSD, for reducing PTSD and depression symptoms among healthcare workers. Primary outcomes improved during the monitoring and intervention phases, although the magnitude of improvement was substantially greater during the intervention period. Among participants above clinical thresholds at baseline, reliable recovery rates increased markedly from the monitoring phase (PTSD: 14.6%; depression: 15.8%) to the intervention phase (PTSD: 77.1%; depression: 68.3%), representing a five-fold increase for PTSD and a threefold increase for depression. These gains were largely sustained at three-month follow-up, with 68.8% of participants maintaining recovery from PTSD and 52.6% from depression. Across the full sample, including participants with subthreshold symptoms, rates of reliable symptom improvement increased substantially during the intervention phase, rising from 41.1% to 65.8% for PTSD and from 20.0% to 63.1% for depression. At three-month follow-up, 72.2% of participants maintained reliable improvement in PTSD symptoms and 56.0% in depression symptoms, indicating sustained therapeutic benefit for individuals above and below clinical thresholds. Mean symptom scores remained below clinical thresholds from post-intervention through three-month follow-up for both PTSD and depression.

Secondary outcomes showed varied patterns. Anxiety symptoms improved during both monitoring and intervention phases, with substantially greater improvement during intervention. In contrast, intervention-specific effects were observed for insomnia severity, resilience, and wellbeing, which showed no significant change during monitoring but significant improvements during the intervention phase. All gains in secondary outcomes were sustained at three-month follow-up. Exploratory analyses of potential cognitive correlates of change revealed that reductions in suppression of unwanted memories and reductions in the strength of posttraumatic cognitions were correlated with PTSD symptom improvement. For depression, reductions in self-devaluative beliefs were associated with symptom improvement, consistent with cognitive models emphasising the role of negative self-beliefs in maintaining depressive symptoms. The improvements in anxiety and insomnia are important given studies show prevalence rates assessed by self-report of 23.2% and 38.9% respectively among healthcare workers^50^, and the established associations with impaired clinical decision-making, medical errors, and burnout^51^. The enhancement of resilience as measured by the CD-RISC, which includes appraisals of perceived coping, suggests that the coaching intervention may have improved perceived coping in relation to occupational stressors or mental health symptoms.

Wellbeing demonstrated a more modest pattern, with planned comparisons revealing intervention-period improvement although the overall effect of time was not significant. This finding may reflect the context in which the study was conducted. Dimensions of wellbeing, such as purpose, self-acceptance, and life satisfaction^52^, may recover more gradually during periods of sustained crisis such as the COVID-19 pandemic, particularly when occupational stressors remain elevated. Collectively, the secondary outcomes indicate meaningful improvements across multiple dimensions of psychological health. However, the presence of monitoring-period change for anxiety (and primary outcomes) necessitates caution in attributing all gains to the SHAPE coaching intervention.

SHAPE coaching demonstrated a very large treatment effect for reductions in PTSD symptoms (partial η² = 0.50), comparable to established trauma-focused treatments. CT-PTSD, the evidence-based treatment upon which SHAPE is based, demonstrates very large within-group treatment effects in routine clinical care (d = 1.88), with 78.8% of patients showing reliable improvement and 57.3% achieving clinically significant change in intent-to-treat analyses^53^. More recently, therapist-assisted internet-delivered CT-PTSD achieved similarly large within-group effects (d = 2.36, 95% CI [2.15, 2.56])^15^ and a controlled effect size compared to wait list of d = 1.67. Compared to waitlist or treatment-as-usual controls, meta-analyses of prolonged exposure report large between-group effect sizes (Hedges’ g = 1.2–1.5), although effects are smaller in military versus civilian populations^54^. For cognitive processing therapy with written trauma accounts in personnel from high-risk occupations (specifically veterans and military personnel), within-group pre-post effects achieve medium effect sizes (g = 0.48)^55^.

The magnitude of change is notable given that SHAPE omitted traditional reliving and prolonged exposure elements. While some non-trauma-focused interventions have shown effects^56^, trauma-focused treatments with memory exposure have a stronger evidence base and demonstrate superior effectiveness in comparison studies^15, 56, 57^. Instead of detailed reliving through imaginal exposure or written trauma narratives, SHAPE utilised Then vs. Now stimulus discrimination as the core memory-focused technique. The intervention targeted naturally occurring triggers and excessively negative cognitions identified through standardised measures, eliminating the need for detailed trauma exploration in coaching calls. Consistent with stimulus discrimination as an early intervention for PTSD^58^, this approach appeared sufficient for reducing intrusive re-experiencing. Combined with coach-delivered rather than specialist-therapist delivery, SHAPE may reduce treatment barriers while maintaining effectiveness, offering a more accessible and scalable pathway to evidence-based care for healthcare workers experiencing PTSD or depression symptoms.

Exploratory analyses of cognitive processes associated with symptom change yielded findings largely consistent with cognitive models of PTSD^25^ and depression^26^. As predicted, reductions in posttraumatic cognitions were significantly associated with PTSD symptom improvement when controlling for baseline severity. This finding aligns with Ehlers and Clark’s cognitive model^25^, which identifies excessively negative trauma-related appraisals (e.g., ‘People are not what they seem,’ ‘I have permanently changed for the worse’) as central maintaining factors in PTSD, and is consistent with prior evidence that changes in these cognitive processes precede symptom reduction with CT-PTSD^28–30^. Similarly, reductions in suppression of unwanted memories were associated with PTSD improvement, supporting the emphasis of the coaching intervention on stimulus discrimination as an alternative response to intrusive memories. Stimulus discrimination helps patients distinguish sensory differences between triggers encountered in the present from those in the past trauma and has been shown to reduce the recurrence of unwanted memories, even when taught prophylactically prior to trauma exposure^59^. These findings suggest that core mechanisms explicitly targeted by the SHAPE intervention, derived primarily from CT-PTSD, may be relevant to symptom change among healthcare workers with trauma-related symptoms. For depression, reductions in self-devaluative beliefs (e.g., ‘I am inadequate,’ ‘There is something wrong with me as a person’) were significantly associated with symptom improvement when controlling for baseline depression severity, consistent with cognitive theories emphasising the role of negative self-beliefs in maintaining depressive symptoms^26^. While these cross-sectional associations are consistent with proposed mechanisms of change, formal mediation analyses with appropriate temporal sequencing are needed to establish whether changes in these cognitive processes mediate symptom improvement.

Contrary to predictions, changes in rumination were not significantly associated with reductions in PTSD or depression symptoms. There are two likely reasons for this unexpected result.  First, the measure used to assess rumination focused on rumination as a response to intrusive memories rather than distinct ruminative styles shown to differentially predict outcomes. Research demonstrates that rumination styles differ in their functional properties and consequences: a concrete, process-focused style (characterised by ‘how’ questions focused on specific contextual details) versus an abstract, evaluative style (characterised by ‘why’ questions focused on meanings and implications of difficulties^60–62^). The abstract style increases over-general memory, impairs problem-solving, and strengthens global negative self-judgments, whereas the concrete style does not produce these deleterious effects^61^. Our measure did not distinguish between these styles. Reductions in abstract rumination, which may have contributed to improvements in depressive symptoms, were not captured by our response-to-intrusions measure. Second, baseline rumination scores were relatively low, with participants reporting infrequent rumination in response to intrusive memories. Scores decreased further during treatment, creating a restricted range that may have produced a floor effect, limiting the variability necessary to detect associations with symptom change. Future research would benefit from more precise measurement of rumination constructs in treatment studies, particularly distinguishing between adaptive and maladaptive ruminative styles or using measures that specifically focus on work-related rumination^63^.

Although statistically significant reductions occurred during monitoring, mean scores remained above clinical thresholds for PTSD, depression, and anxiety. Rates of reliable recovery and remission during monitoring were modest, with moderate rates of reliable improvement. In contrast, symptom improvement during intervention was statistically significant and clinically meaningful, with high rates of reliable improvement, remission, and recovery. For PTSD, recovery rates were five times greater during intervention than during monitoring; for depression, reliable recovery rates were three times greater. Gains were sustained at three-month follow-up, with mean scores below clinical thresholds on all symptom measures. The assessment and monitoring period was employed as a comparison, however, individuals reaching this point had received rapid assessment and were also informed that they would be able to access the intervention in a short time (three weeks, as opposed to months or years). Often, the practitioner completing the assessment also delivered the intervention as opposed to assessments being conducted by independent assessors. It is possible that this led to higher levels of hope and expectancy which precipitated greater change during this period than would be seen in a randomised trial with an independent assessor and waitlist. A randomised controlled trial and follow-up is required.

The six-session intervention evaluated herein was shorter than the eight to twelve, sixty-to-ninety-minute sessions recommended as the first-line treatment for PTSD. Consistent with our rationale that a digitally enabled coaching intervention may improve accessibility for healthcare workers through reducing the burden of time, SHAPE required a relatively low overall time commitment compared with other evidence-based PTSD interventions. Participants engaged in approximately six hours of combined coaching and self-guided module completion across six weeks, equivalent to roughly 20–80 min per week. In practice, standard face-to-face trauma-focused therapies often involve 12–18 h of therapist contact plus homework. In a trial of a therapist-guided digital intervention, iCT-PTSD^15^, participants spent an average of 24.8 h engaging with the programme, with therapists providing an additional 6–7 h of contact time. The RESTORE intervention^7^ developed for healthcare workers in the pandemic involved 8 self-study modules, anticipated to require 30–40 min. Unguided interventions are likely to require less time, but may not be suited to those presenting with diagnostic PTSD. The relatively brief and structured but still guided format of SHAPE may therefore enhance feasibility and accessibility for this workforce, while maintaining comparable clinical outcomes to existing evidence-based PTSD interventions.

Several factors may have contributed to the recovery rates including timely access, ease of access, and treatment preference. Time spent waiting can be associated with treatment outcomes^64, 65^ and research suggests that less than half of individuals with depression demonstrate a preference for in-person therapy^66^. Participants in this intervention could take part wherever they were able to get private access to a telephone and, often, healthcare workers chose to take the calls while at work. There is evidence that receiving a preferred treatment is linked to better outcomes^67^. Participants in this study who were above clinical cut offs were offered the choice of coaching or a referral to their local NHS talking therapies, thus each individual stated a preference for coaching and was able to move from assessment to intervention in three weeks. Since all individuals participating in this study were healthcare professionals, the sample is likely to be more homogenous than a general population mental health sample. Healthcare workers may benefit especially from a cognitive therapy coaching approach. This may be due to education level, stable employment, awareness of health and wellbeing, and motivation. The coaching sessions required participants to consolidate learning of new behaviours independently to sessions, requiring a significant level of motivation which has been linked to outcomes^68^.

Results suggest greater recovery in PTSD than depression symptoms, possibly reflecting that intervention targets were derived from CT-PTSD rather than first-line depression treatments. Although delivered during the pandemic peak, the impact of COVID-19 on mental health appeared to exacerbate pre-existing issues rather than constitute the primary concern. Index traumas were largely unrelated to COVID-19, with 76.9% of participants with clinically significant symptoms reporting unrelated trauma and 55.4% bringing personal rather than occupational trauma to intervention. Childhood trauma was reported by 35%, replicating high childhood adversity rates in paramedic samples^1, 2^ and suggesting this may be a broader workforce issue, especially among those presenting with mental health difficulties.

In summary, this cognitive therapy coaching intervention was associated with greater reductions in PTSD and depression symptom severity when compared to a monitoring-only period. Correlates of clinical outcome derived from cognitive conceptualisations of PTSD and depression were related to symptom reduction: reductions in suppression in response to unwanted memories and posttraumatic cognitions, as specified in Ehlers and Clark’s cognitive model of PTSD persistence^25^, significantly correlated with PTSD symptom reduction whilst changes in self-devaluative appraisals correlated with reductions in depression symptom severity.

### Limitations

This was not a randomised controlled trial. Although symptom improvements during the intervention period exceeded those during monitoring, and recovery rates were substantial, the effects of time and natural recovery cannot be definitively excluded. Participants demonstrated statistically significant improvements during the three-week monitoring period, consistent with research demonstrating beneficial effects of symptom monitoring for some patients with PTSD^69, 70^ and with natural recovery trajectories. Some intervention-phase gains may reflect continuation of this trajectory. However, several findings support intervention-specific effects: reliable recovery rates increased five-fold for PTSD and three-fold for depression during the intervention phase, disproportionate to the difference in phase duration; effect sizes were substantially larger during intervention than monitoring; and insomnia, resilience, and wellbeing showed no change during monitoring but significant improvement during intervention. Furthermore, with a median time since trauma of 18 months, participants were well beyond the acute phase when spontaneous recovery is most common. Nevertheless, a randomised controlled trial with an appropriate comparison condition is essential to unambiguously establish efficacy.

Our correlational analyses examining associations between changes in cognitive processes and reductions in symptoms cannot establish temporal precedence or causal relationships. While this approach provides preliminary evidence of covariation between cognitive processes and symptom change, future studies employing mediation analyses with larger sample sizes and more frequent measurement points are needed to test whether changes in these processes precede symptom improvement.

Our sample was heterogeneous with respect to trauma exposure and symptom severity, reflecting our inclusive recruitment strategy. This approach prioritised accessible support for healthcare workers experiencing trauma-related or depression-related distress during the COVID-19 pandemic. While heterogeneity enhances the ecological validity and generalisability of our findings to diverse healthcare populations, it may limit comparisons to trials with more restrictive inclusion criteria. Additionally, most participants remained in active employment throughout the study, indicating maintained occupational functioning despite symptom severity. Whether brief cognitive therapy coaching would be sufficient for more severely impaired individuals or specific clinical presentations, such as PTSD with high levels of dissociation, remains unclear and warrants investigation in future trials.

The sample was predominantly female, consistent with the composition of the NHS workforce. Future research should explore barriers to access for minority groups and underrepresented professions. This study assessed intervention acceptability through monitoring attrition only (*n* = 4, 3.9%). Treatment fidelity was monitored through supervision and session documentation procedures. Future studies should incorporate comprehensive acceptability measures and independent ratings of treatment fidelity to complement the approach employed in this study.

## Conclusions

Healthcare workers are a group with elevated risk for depression and PTSD. Despite this increased risk, there are few specialist, evidence based and accessible interventions available for them. The COVID-19 pandemic represented a global health emergency that drew attention to and appeared to exacerbate the pre-existing mental health needs of this population. This pilot evaluation study demonstrates that cognitive therapy coaching represents an effective and promising intervention. Future work will comprise a randomised controlled trial to address the confound of natural recovery over time, the acceptability of the intervention, health economics, and barriers to access for underrepresented populations.

## Supplementary Information

Below is the link to the electronic supplementary material.

Supplementary Material 1

## Data Availability

The datasets generated and analysed in the current study are available from the corresponding authors on reasonable request.
