# Supplementary material for: Evaluation of SHAPE cognitive therapy coaching for PTSD and depression symptoms in healthcare workers repeatedly exposed to trauma
Source: Sci Rep. 2026 Jan 17;16:5593. doi: 10.1038/s41598-026-36057-5 (PMC12891615; doi:10.1038/s41598-026-36057-5)
Supplement: Supplementary file 1 — Supplementary Material 1 [file 41598_2026_36057_MOESM1_ESM.docx]

**Supplementary Material 1**

Table of Contents

[Supplement 1: Core differences in trauma-focused trigger discrimination in SHAPE vs CT-PTSD 2](#_Toc211426643)

[Supplementary Table S1: Example of Then vs Now trigger discrimination in SHAPE coaching 3](#_Toc211426644)

[Supplementary Table S2: Core Tools Used in SHAPE Weekly Calls 4](#_Toc211426645)

[Supplementary Table S3: SHAPE Online Learning Modules 5](#_Toc211426646)

**Supplementary Information**

# **Supplement 1:** Core differences in trauma-focused trigger discrimination in SHAPE vs CT-PTSD

In **CT-PTSD and trauma-focused CBT for PTSD therapy in general,** exposure to triggers is deliberate and therapist-initiated after triggers have been identified from discussion of situations where the patient recently had re-experiencing symptoms. In CT-PTSD, this discussion includes identifying similarities and differences between the trigger and its context and the corresponding stimulus in the context of trauma. This leads to the conclusion that there were many more differences than similarities and the patient was safe. Discrimination is then practiced by bringing a relevant trigger into the therapy session and asking the patient to focus on all the differences between the trigger in the present context and the trauma. This is different from traditional exposure which focuses on prolonged exposure to the feared stimuli. In CT-PTSD, for example, a therapist might deliberately play the beeping sound of a heart monitor in session to trigger a trauma memory in which a heart monitor was present, then guide the patient to discriminate between the audio sound of the monitor in session (the trigger) and the past trauma in which the monitor was beeping. Following this practice in the session, the patient then practices applying the technique to triggers in their everyday life. In contrast, SHAPE coaching does not include practicing discrimination with triggers in the session. Instead, the coach guides the patient through a trigger discrimination exercise for a trigger they encountered in their daily lives (e.g., encountering a patient with similar injuries, hearing an alarm). Participants are encouraged to approach rather than avoid naturally occurring triggers and apply stimulus discrimination, distinguishing between the trigger in the present safe context and the past trauma.

SHAPE extends the CT-PTSD stimulus discrimination (Then vs Now) technique by incorporating new information in the discrimination procedure that targets excessively negative appraisals associated with the trigger. These appraisals are identified by the posttraumatic cognitions inventory (PTCI; Foa et al., 1999) rather than deliberately exploring the meaning of the trauma in coaching calls. For example, perhaps a healthcare worker’s trauma relates to a patient who died with respiratory illness when she asked to take a break while feeling overwhelmed. Family members who visited wore face masks. Perhaps masks worn by a shop assistant today trigger the memory of the patient who passed away and self-evaluations of inadequacy (measured on the PTCI) for taking a break. The Then vs Now discrimination the coach may conduct with the healthcare worker will first note the similarities and the differences in the sensory features of the trigger today and the past trauma. The coach would then go onto to include new information that the healthcare worker has now accessed through surveys organised by the coach, which they did not have then.

Supplementary Table S1 provides an example of Then vs Now trigger discrimination used in SHAPE coaching.

#

# **Supplementary Table S1**: Example of Then vs Now trigger discrimination in SHAPE coaching

| **Then** | **Now** |
| --- | --- |
| **Similarities** | |
| Person with brown hair and brown eyes walking towards me with mask | Person with brown hair and brown eyes walking towards me with mask |
| Feel apprehensive | Feel apprehensive |
| The mask is black | The mask is black |
| I am standing with my arms stretched out in front of me | I am standing with my arms stretched out in front of me |
| Fluorescent overhead lighting | Fluorescent overhead lighting |
|  |  |
| **Differences** |  |
| Person is a family member | Person is a shop assistant |
| I am attending to a patient | I am shopping for dinner items |
| My arms are stretched in front of me, holding the patient’s bed | My arms are stretched in front of me holding the shopping trolley |
| A patient is laying in a hospital bed | There is no one laying down |
| Two people in the room | Several people walking around the shop |
| I believed I was inadequate for having to take a break and ask the head nurse to take over | I now know that taking a break was absolutely necessary to continue to care for the whole range of patients being admitted. A survey that my coach and I put together, completed by 6 nurses that she contacted, revealed that none of them rated me as inadequate. They described me as competent, caring and working well under extreme conditions. (new information) |

Supplementary Table S2 lists the core tools administered in SHAPE and Supplementary Table S3 outlines the available modules.

# **Supplementary Table S2**: Core Tools Used in SHAPE Weekly Calls

| Core Tools |
| --- |
| 1. THEN vs. NOW for re-experiencing symptoms |
| 1. IF THEN plan for rumination |
| 1. Planning ahead for low mood and avoidance |
| 1. Updating unhelpful thoughts with surveys and/or behavioural experiments |
| 1. Responsibility pie charts for guilt and blame |
| 1. Realistic risk and worry diary for worry |

#

# **Supplementary Table S3:** SHAPE Online Learning Modules

|  | **SHAPE Modules** |
| --- | --- |
| **1** | **Module:** It Matters What you Focus On: Helpful and Unhelpful Attention |
|  | **Primary Focus:** Self-focused attention  **Intervention exercises:** Two healthcare workers explain how externally-focused attention helps them, behavioural experiment to test the effects of shifting focus of attention, Attention Gym with 2 x 5 mins attention training exercises.  **Tools:** (1) Behavioural experiment to test predictions associated with feeling self-conscious; (2) Four steps to shift from self-to-externally focused attention |
| **2** | **Module:** Get Out of Your Head with Helpful Thinking |
|  | **Primary Focus:** Updating negative appraisals related to low resilience  **Intervention exercises:** Identifying extreme thinking, exercise to update extreme thoughts linked to low resilience (negative self-beliefs), exercise to elicit the effects of flexible and extreme thinking, audio exercise to elicit the effects of abstract vs practical thinking, video exercise applying practical thinking to critical incidents.  **Tools:** (1) Three steps to elicit practical thinking, (2) Behavioural experiment to update negative self-belief(s) |
| **3** | **Module:** Habits and Dwelling: How to Change Them |
|  | **Primary Focus:** Rumination  **Intervention exercises:** Identifying dwelling with three questions, video illustration of how habits develop (through associative learning), video testimony of a paramedic describing his warning signs for dwelling, exercise to elicit IF-THEN plan to apply to warning signs for rumination.  **Tool:** (1) IF-THEN plan |
| **4** | **Module:** Dealing with Unwanted Memories: Then vs Now |
|  | **Primary Focus:** Intrusive memories (including rumination as a response)  **Intervention exercises:** Video testimony (how two paramedics deal with unwanted memories), video exercise to elicit the effects of suppression, how to use Then vs Now to triggers in the present that overlap with stimuli from past trauma or critical incidents, practice exercise with video clips and audio triggers  **Tools:** (1) Then vs Now trigger discrimination |
| **5** | **Module:** Transforming Worries and Improving Performance |
|  | **Primary Focus:** Worry  **Intervention exercises:** Video testimony (how two healthcare workers deal with worries), worry diary to determine the percentage of worries that come true, calculating realistic risk for worries, behavioural experiment to test worry predictions, imagery exercise to focus on the likely (rather than catastrophic) outcome, planning ahead exercise. **Tools:** (1) Worry diary (2) Realistic risk and (3) Planning ahead. |
| **6** | **Module:** Dealing with Guilt and Self-Blame |
|  | **Primary Focus:** Guilt and self-blame **Intervention exercises:** Cognitive restructuring using: (1) Responsibility pie chart for guilt/self-blame (2) Hindsight bias correction (distinguishing what you knew then vs. now) (3) Remembering reasons for behaviour during the event (4) Recognising helpful actions taken (5) Considering realistic alternative outcomes (6) Challenging emotional reasoning (7) Identifying superhuman standards  **Tools:** Responsibility pie chart for guilt/self-blame |
